# A Long-Chain Dextran Produced by *Weissella cibaria* Boosts the Diversity of Health-Related Gut Microbes Ex Vivo

**DOI:** 10.3390/biology13010051

**Published:** 2024-01-18

**Authors:** Maria Tintoré, Jordi Cuñé, Lam Dai Vu, Jonas Poppe, Pieter Van den Abbeele, Aurélien Baudot, Carlos de Lecea

**Affiliations:** 1 AB Biotek Human Nutrition and Health, Peterborough PE7 8QJ, UK; 2 Cryptobiotix SA, Technologiepark-Zwijnaarde 82, 9052 Ghent, Belgium; lamdai.vu@cryptobiotix.eu (L.D.V.);

**Keywords:** prebiotic, long-chain dextran, ex vivo, *Bifidobacterium*, *Bacteroides*, propionate, butyrate, gas production, microbial diversity, gut microbiome, preclinical research

## Abstract

**Simple Summary:**

Conventional diversity metrics do not fully capture treatment impacts on microbial diversity. An innovative community modulation score (CMS), coupled with the predictive SIFR® technology, underlined the potential of a bacterial long-chain dextran as a booster of microbial diversity, as compared to the well-established prebiotic inulin.

**Abstract:**

Long-chain dextrans are α-glucans that can be produced by lactic acid bacteria. NextDext^TM^, a specific long-chain dextran with a high degree of polymerisation, produced using *Weissella cibaria*, was recently shown to exert prebiotic potential in vitro. In this study, the ex vivo SIFR^®^ technology, recently validated to provide predictive insights into gut microbiome modulation down to the species level, was used to investigate the effects of this long-chain dextran on the gut microbiota of six human adults that altogether covered different enterotypes. A novel community modulation score (CMS) was introduced based on the strength of quantitative 16S rRNA gene sequencing and the highly controlled ex vivo conditions. This CMS overcomes the limitations of traditional α-diversity indices and its application in the current study revealed that dextran is a potent booster of microbial diversity compared to the reference prebiotic inulin (IN). Long-chain dextran not only exerted bifidogenic effects but also consistently promoted *Bacteroides* spp., *Parabacteroides distasonis* and butyrate-producing species like *Faecalibacterium prausnitzii* and *Anaerobutyricum hallii*. Further, long-chain dextran treatment resulted in lower gas production compared to IN, suggesting that long-chain dextran could be better tolerated. The additional increase in *Bacteroides* for dextran compared to IN is likely related to the higher propionate:acetate ratio, attributing potential to long-chain dextran for improving metabolic health and weight management. Moreover, the stimulation of butyrate by dextran suggests its potential for improving gut barrier function and inflammation. Overall, this study provides a novel tool for assessing gut microbial diversity ex vivo and positions long-chain dextran as a substrate that has unique microbial diversity enhancing properties.

## 1. Introduction

The gut microbiota, consisting of trillions of microbial cells, impacts human health [1]. It plays a pivotal role in maintaining intestinal barrier integrity [2,3,4], gut–brain communication [5] and modulating adaptive immunity [6]. Consequently, aberration in gut microbiota composition has been associated with a range of diseases [1]. Gut microbes ferment dietary components and produce metabolites, which impact their host [7]. Short-chain fatty acids (SCFA, mainly acetate, propionate and butyrate) are among the most studied microbial metabolites and have been linked to health benefits [8]. While acetate production pathways are widely distributed, the pathways for propionate and butyrate production are limited to certain species, such as members of *Bacteroidaceae* (propionate), *Lachnospiraceae* and *Ruminococcaceae* (butyrate) [9,10]. Further, *Bifidobacterium* spp., unable to produce butyrate, have also been shown to indirectly stimulate butyrate in a process called cross-feeding [11,12,13]. These taxa are thus interesting targets for dietary supplements and therapeutics that aim to improve gut health. 

Prebiotics are substrates that remain intact as they pass through the upper gastrointestinal tract and are then selectively utilized by host micro-organisms in the colon, thus eliciting health benefits [14,15]. Many (potential) prebiotics are carbohydrates that can originate from algae (e.g., alginates, fucoidans) [16], plants (e.g., fructooligosaccharides, inulin) [17] or animals (e.g., chitosan) [18]. Further, Khan et al. (2022) recently reviewed another class of potential prebiotics, i.e., bacterial polysaccharides [19]. An example of this class are dextrans, α-glucans produced by the majority of lactic acid bacteria [20,21,22,23]. Being homopolysaccharides (exclusively consisting of glucose), dextrans structurally differ from established prebiotics. Dextrans consist of α-(1→6) bonds and adopt a helical shape, substituted with α-(1→2), α-(1→3) or α-(1→4) branches. The degree of polymerization (DP) and branching patterns strongly depend on the bacterial strain used for its production [24]. Bacterial polysaccharides provide advantages such as purity, hydrophilicity, and suitability for large-scale industrial production. Moreover, recent preliminary in vitro studies attributed prebiotic potential to dextrans [20,22]. Nevertheless, microbiome-wide effects have yet to be investigated and it remains unclear how the effects of dextrans differ from those of reference prebiotics such as inulin (IN) [25]. 

In vitro gut models have the potential to complement human studies by reducing confounding factors such as dietary patterns and transit time [26,27]. However, in vitro gut models often suffer potential composition bias due to drastic differences between in vivo-derived and lab-colonizing microbiota; short-term gut models are favorable for fast-growing, aerotolerant taxa [28,29,30,31], while long-term gut models enriched taxa that thrive under very defined nutritional and environmental conditions [32,33]. In addition, the low throughput of in vitro models hinders their ability to address interindividual differences. In contrast, the recently developed ex vivo SIFR^®^ technology (Systemic Intestinal Fermentation Research), a high-throughput bioreactor-based technology, enables the inclusion of multiple test subjects in the study design, which provides predictive insights (within 1–2 days) for the outcomes of clinical studies performed over weeks of intervention [34]. 

In this study, the SIFR^®^ technology was used to investigate the effects of the high DP dextran NextDext^TM^, produced using a wild-type strain of *Weissella cibaria*, on gut microbial composition of healthy human adults (*n* = 6). In addition, the production of SCFA, branched chain fatty acid (bCFA) and gasses was also assessed. Treatment with the reference prebiotic IN was included for side-by-side comparison. Additionally, based on the high accuracy of quantitative sequencing to quantify density of bacteria and the exactly known incubation volumes when assessing changes in microbial composition using the SIFR^®^ technology, a novel diversity index was introduced, i.e., the community modulation score (CMS). The CMS represents either the number of species that increased (positive CMS) or decreased (negative CMS) upon treatment. Further, the combined CMS has a positive value when the number of increased species exceeds the number of decreased species, suggesting that treatment overall enhances microbial diversity.

## 2. Materials and Methods

### 2.1. Test Compounds

The test compounds were IN from chicory (I2255, Merck, Overijse, Belgium), and NextDext^TM^ (AB Biotek HNH, Barcelona, Spain). IN was included as a reference prebiotic and is a polymer of β(2,1)-bond-linked fructose residues with a chain-terminating glucose with an average fructose:glucose ratio of 20:1 (DP = 20 (on average)). NextDext^TM^ is a food-grade native homopolysaccharide with high DP (DP > 11000). This α-glucan is obtained through fermentation from sucrose as a carbon source by the NCIMB 42196 strain. The production process is described in the Patent PCT/EP2014/000360 [20]. While Amaretti et al. (2020) [20] already demonstrated that this substrate could have a differential prebiotic effect with traits beyond bifidogenic effects, this previous study was limited in terms in terms of resolution of the techniques employed. 

### 2.2. SIFR^®^ Technology

The SIFR^®^ technology was developed to study the human gut microbiota in a highly biorelevant manner across numerous parallel test conditions (both treatments and test subjects) [34]. Briefly, individual bioreactors were processed in a bioreactor management device (Cryptobiotix, Ghent, Belgium). Each bioreactor contained 5 mL of a nutritional medium–faecal inoculum blend supplemented with 5 g of the test compound/L, then sealed individually, before being rendered anaerobic. Blend M0017 was used for the preparation of the nutritional medium (Cryptobiotix, Ghent, Belgium). After preparation, bioreactors were incubated under continuous agitation (140 rpm) at 37 °C (MaxQ 6000, Thermo Scientific, Thermo Fisher Scientific, Merelbeke, Belgium).

**Figure 1:**
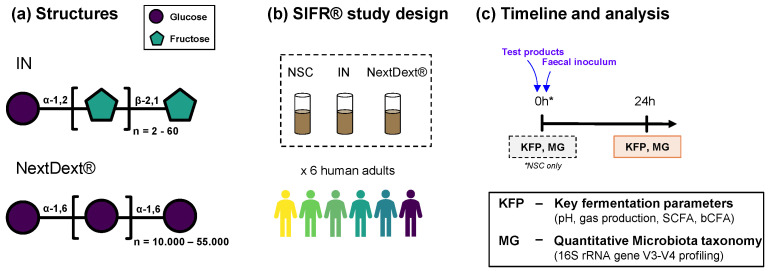
**Study design using the ex vivo SIFR^®^ technology to assess the effect of dextran on the gut microbiota of human adults (*n* = 6).** (**a**) Chemical structures of the test products. (**b**) Reactor design using the ex vivo SIFR^®^ technology to evaluate the impact of dextran at an equivalent dose of 5 g/d compared to the reference prebiotic IN (5 g/d) and a reference without additional substrate (NSC). (**c**) Timeline and analysis at different time points. * refers to analysis in the control arm (NSC).

Three experimental conditions were tested for 6 human adults: a no-substrate control (NSC), 5 g/d inulin (IN), and 5 g/d dextran (Figure 1). For each of the 6 faecal samples, this NSC incubation was initiated simultaneously, consisting of an optimized nutritional medium and microbiota without a test product. The advantage of comparing test products to NSC is that any changes between the NSC and test products can solely be attributed to the addition of the test products. Following 24 h incubation, the pressure was measured in the bioreactors’ headspace, and liquid samples were subsequently collected for the analysis of key fermentation parameters and microbial composition. This time point was used as prebiotic effects at 24 h in the SIFR^®^ technology have been shown to correspond to findings of clinical studies where such prebiotic substrates were administered over a period of weeks [34]. 

Fresh faecal samples were collected according to a procedure approved by the Ethical Committee of the University Hospital Ghent (reference number BC-09977). This procedure required participants to sign informed consent in which they donated their faecal sample for the current study. The selection criteria for the 6 donor samples used herein were as follows: 25–65 years of age, no antibiotic use in the past 3 months, no gastrointestinal disorders (cancer, ulcers, IBD), no use of probiotic, non-smoking, alcohol consumption < 3 units/d and BMI < 30. These criteria were based on observations of the Belgian Flemish Gut Flora Project where deviations from the aforementioned criteria were shown to contribute to variation in gut microbiome composition [35]. For this specific study, 3 male and 3 female donor samples were assessed (average age = 41.0 years). 

### 2.3. Key Fermentation Parameters

SCFA (acetate, propionate, butyrate, and valerate) and bCFA (sum of isobutyrate, isocaproate, and isovalerate) were extracted with diethyl ether. Briefly, 0.5 mL samples were diluted in distilled water (1:3), acidified with 0.5 mL of 48% sulfuric acid, after which an excess of sodium chloride was added along with 0.2 mL of internal standard (2-methylhexanoic acid) and 2 mL of diethyl ether. Upon homogenization and separation of the water and diethyl ether layer, diethyl ether extracts were collected and analysed using a Trace 1300 chromatograph (Thermo Fisher Scientific, Merelbeke, Belgium) equipped with a Stabilwax-DA capillary GC column, a flame ionization detector, and a split injector using nitrogen gas as the carrier and makeup gas. The injection volume was 1 µL and the temperature profile was set from 110 °C to 240 °C. The carrier gas was nitrogen, and the temperatures of the injector and detector were 240 and 250 °C, respectively. Sample pH was measured using an electrode (Hannah Instruments Edge HI2002, Temse, Belgium).

### 2.4. Microbiota Phylogenetic Analysis: Quantitative 16S rRNA Gene Profiling

Quantitative data were obtained by correcting abundances (%; 16S rRNA gene profiling) with total cell counts (cells/mL; flow cytometry), resulting in the estimated absolute cell counts per mL of different taxonomic groups. Initially, a bacterial cell pellet was obtained by the centrifugation of 1 mL samples for 5 min at 9000× *g*. DNA was extracted via the SPINeasy DNA Kit for Soil (MP Biomedicals, Eschwege, Germany), according to the manufacturer’s instructions. Subsequently, library preparation and sequencing were performed on an Illumina MiSeq platform with v3 chemistry. The 16S rRNA gene V3–V4 hypervariable regions were amplified using primers 341F (5′-CCT ACG GGN GGC WGC AG-3′) and 785Rmod (5′-GAC TAC HVG GGT ATC TAA KCC-3′). The results were analysed at different taxonomic levels (phylum, family, and operational taxonomic unit (OTU) level). 

For the total cell count analysis, liquid samples were diluted in anaerobic phosphate-buffered saline (PBS), after which cells were stained with SYTO 16 at a final concentration of 1 µM and counted via a BD FACS Verse flow cytometer (BD, Erembodegem, Belgium). Data were analysed using FlowJo, version 10.8.1.

### 2.5. Diversity Indices

α-diversity (species richness and species evenness) was estimated via the observed number of OTUs, the Chao1 index, the reciprocal Simpson diversity index and Shannon diversity index. These indices reflect species richness (e.g., observed number of species and the Chao1 diversity index) and/or evenness (e.g., reciprocal Simpson diversity and Shannon diversity index), two fundamentally different concepts. While species richness is higher as more taxa are present, species evenness is higher as taxa are more evenly distributed.

In addition, a novel community modulation score (CMS) was introduced based on the strength of quantitative sequencing to provide quantitate insights and thus (unlike proportional insights) evaluate whether microbial taxa truly increased upon treatment. In short, the community modulation score (CMS) represents the number of OTUs (out of the 100 most abundant ones) that increased (positive CMS) or decreased (negative CMS) upon treatment. The combined CMS has a positive value when the number of increased species exceeds the number of decreased species, suggesting that the treatment is a diversity booster. The community modulation score is based on the assumption that an OTU has increasingly or decreasingly grown upon treatment with a specific substrate when its levels increased or decreased with more than 30% compared to the NSC, respectively:

$$Positive\;CMS=\sum_{x=1}^{100}\left(({OTUx}_{treatment}>{OTUx}_{NSC}\times 130\%\;)\Rightarrow \;1\right)$$

$$Negative\;CMS=\sum_{x=1}^{100}\left(\left({OTUx}_{NSC}>{OTUx}_{Treatment}\times 130\%\;\right)\Rightarrow -1\right)$$

Combined CMS=Positive CMS+Negative CMS

The 30% threshold is based on historical data that 15% is the technical variation (standard deviation) in OTU detection via quantitative sequencing in different biological replicates of SIFR^®^ bioreactors (internal data) so that an increase with 30% (=2 times the standard deviation), according to univariate statistical tests, indeed provides 95% certainty that an OTU truly increased upon treatment. Technical variation for species-level detection via shotgun sequencing was recently shown to be 15.2% for different biological replicates of SIFR^®^ bioreactors, thus further corroborating the 15% rule-of-thumb for species/OTU-level detection via quantitative sequencing in SIFR^®^ bioreactors [34].

### 2.6. Statistical Analysis

All univariate and multivariate analyses were performed using R (version 4.2.2; www.r-project.org; accessed on 28 October 2023). For the principal component (PCA) analysis, the FactoMineR package was used [36]. Regularized Canonical Correlation Analysis (rCCA) was executed using the mixOmics package with the shrinkage method for estimation of penalization parameters (version 6.20.3) [37]. Significance of the supplementation effects compared with the NSC were assessed via repeated measure ANOVA analyses (based on paired testing among the 6 human adults) using the rstatix package, with *p*-value-correction according to Benjamini–Hochberg [38,39]. Taxa that were not significantly affected were further assessed for consistent changes. To be considered as consistently increasing/decreasing for either treatment, taxa had to be present in at least four out of six test subjects and consistently increasing or decreasing for all the test subjects where the taxa were detected.

All visualizations in R were enhanced using the ggplot2 package [40]. For analysis of microbial composition, three measures were taken. First, the statistical analysis was performed on the log_10_-transformed values. Second, a value of a given taxonomic group below the limit of detection (LOD) was considered equal to the overall LOD according to the procedure elaborated by Van den Abbeele et al. (2023) [34]. Finally, a threshold was set to retain the 100 most abundant OTUs in the analysis, to avoid excessive *p*-value corrections.

## 3. Results

### 3.1. Microbiota of Six Human Adults Cover Clinically Relevant Interpersonal Differences

**Figure 2:**
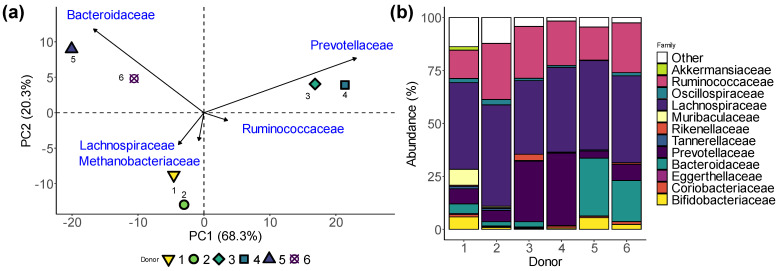
**Microbiota of six human adult donors cover clinically relevant interpersonal differences.** (**a**) PCA based on centred abundances at the family level (%) demonstrating the variation across the faecal microbiota of the human adults. (**b**) Abundances (%) of the key families of the six faecal microbiota.

The composition of the faecal microbiota (used to inoculate SIFR^®^ bioreactors) exhibited marked differences among the six tested human adults (Figure 2). Key differences were either high *Prevotellaceae* levels (donors 3/4), high *Bacteroidaceae* levels (donors 5/6) or high *Lachnospiraceae* and *Methanobacteriaceae* levels (donors 1/2). The stratification of human adults according to these families is in line with the classification of human adult microbiota according to gut enterotypes [41]. The representation of key enterotypes by the six human adults suggests that the test subjects included in the current study cover key interpersonal differences in gut microbiota composition observed in vivo.

### 3.2. Dextran Stimulated the Growth of Human Adult Gut Microbiota Ex Vivo

**Figure 3:**
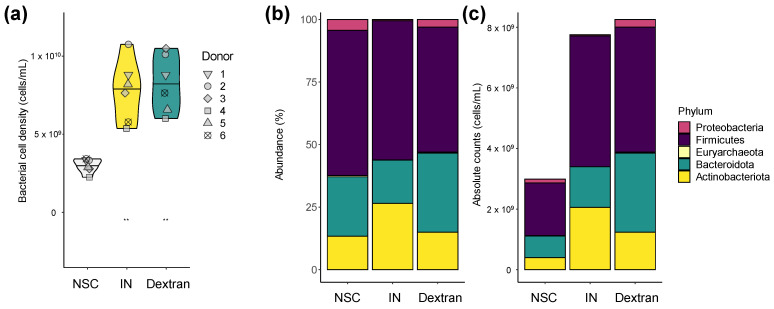
**Dextran and IN stimulated growth of human adult gut microbes ex vivo.** (**a**) Bacterial cell density (cells/mL) of microbial communities derived from human adults (*n* = 6) as tested via the ex vivo SIFR^®^ technology upon treatment with dextran and IN. Statistical differences between treatments and the NSC are indicated with ** (0.001 < p_adjusted_ < 0.01). (**b**) Microbial composition (phylum level) presented as proportional values (%), averaged across the six human adults evaluated. (**c**) Microbial composition presented as absolute values (cells/mL). Briefly, these quantitative insights were obtained by multiplying proportional values (%, shown in (**b**)), with total cell counts (cells/mL, shown in (**a**)) for each individual sample, after the average across the 6 test subjects within a test condition was calculated, as presented in figure (**c**).

Dextran and IN increased bacterial cell density compared to the NSC at 24 h, suggesting that like IN, dextran is used by gut microbes as a substrate for growth (Figure 3a). Due to the significant differences in cell numbers among samples, proportional data obtained via sequencing (in %, Figure 3b) were normalized to more accurately assess changes in microbial composition upon treatment (Figure 3c). The importance of this correction followed from the observation that based on proportional data, dextran did not impact Actinobacteriota (containing *Bifidobacteriaceae* family), while quantitative data revealed a marked increase in this phylum by dextran. Subsequent analysis of microbial composition relies exclusively on quantitative insights.

### 3.3. Dextran Exhibited Prebiotic Effects on Species Richness and Evenness of the Gut Microbiota According to Traditional α-Diversity Indices

**Figure 4:**
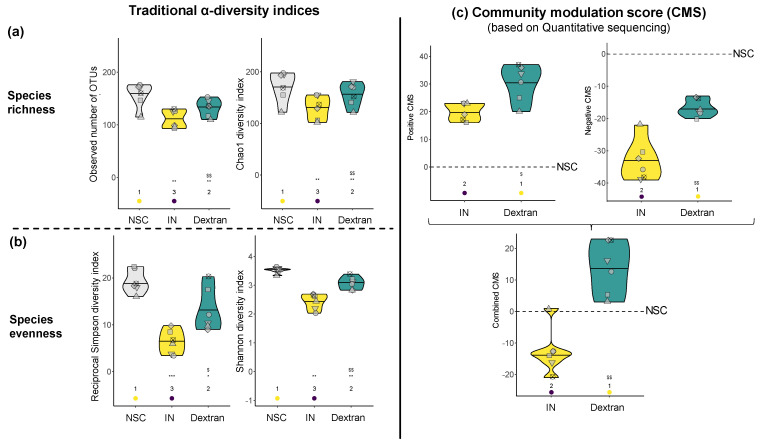
**Dextran supported the high microbial diversity of the human adult gut microbiota ex vivo**. The impact of dextran and IN on traditional α-diversity indices calculated based on OTUs, focusing on (**a**) species richness (observed number of OTUs, Chao1 index) and (**b**) species evenness (reciprocal Simpson diversity index, Shannon diversity index) and (**c**) the novel community modulation scores (CMS), presented as a positive (increased OTUs), negative (decreased OTUs) and combined score. Statistical differences between treatments and the NSC are indicated with * (0.01 < p_adjusted_ < 0.05), ** (0.001 < p_adjusted_ < 0.01) or *** (p_adjusted_ < 0.001), while differences between dextran and IN are indicated with $/$$ (0.01–0.05/0.001–0.01).

The untreated parallel test arm (NSC) simulates the consumption of a diverse diet and thus supports high microbial diversity. Given the inherently high diversity in this NSC, it was crucial to include a reference prebiotic (IN) to effectively evaluate the impact of test products (dextran) on diversity. To gain comprehensive insights, four traditional α-diversity indices were calculated. First, when focusing on species richness (Figure 4a), the observed number of OTUs and Chao1 index were found to be significantly higher for dextran compared to IN. When also accounting for species evenness, diversity markedly decreased for both treatments compared to NSC (Figure 4b). This reflects a less even distribution among dominant gut microbes, thus suggesting that dextran and IN selectively increased specific gut microbes or, in other words, that they were selectively fermented by specific gut microbes, in line with the prebiotic definition [15]. Nevertheless, dextran had a significantly lower impact on species evenness than IN, suggesting that the stimulated gut microbes are more evenly stimulated in response to dextran compared to IN.

### 3.4. Considerations on Limitations and Interpretation of Outcomes of Traditional Diversity Indices

Combining sequencing data with bacterial cell density provided insights into the limitations of traditional α-diversity indices. First, these indices rely on sequencing of the DNA of only the most abundant species. During the current project, averages of 18,197, 32,779 and 25,621 reads were obtained for NSC, IN and dextran samples, respectively. Diversity indices thus rely on the sequencing of DNA belonging to cells that are more abundant than 0.006%, 0.003% or 0.004% in NSC, IN and dextran samples, respectively (=one read/total number of reads; assuming one 16S rRNA gene copy per cell). Given the average respective cell densities of 3.0 × 10^9^, 7.8 × 10^9^ and 8.3 × 10^9^ cells/mL, a bacterial species should, on average, be more abundant than 1.8 × 10^4^ (=0.006% of 3.0 × 10^9^), 2.3 × 10^4^ and even 3.3 × 10^4^ cells/mL, in order to be detected in the NSC, IN and dextran samples, respectively (exact limit of detection for each sample was plotted in Figure S1). The depth at which a community is analysed is thus larger for low-abundance communities (e.g., lower LOD for NSC) as opposed to high-abundance communities (e.g., high LOD for IN and dextran). As a result, upon treatment with test products that increase cell density (e.g., IN or dextran), it becomes more difficult to detect low-abundance species. A lower species richness upon prebiotic treatment should thus be interpreted with caution as it could simply reflect a higher LOD upon treatment.

### 3.5. The Novel Community Modulation Score Shows That Dextran Supported a High Microbial Diversity 

Based on these limitations, a novel community modulation score (CMS) was implemented. The CMS uses the strength of quantitative sequencing and estimates the number of species that increased (positive CMS) or decreased (negative CMS) in the presence of a test product (out of the 100 most abundant OTUs). Interestingly, both the positive CMS and negative CMS were higher for dextran compared to IN treatment (Figure 4c). The combined CMS was positive for dextran (13.8) and negative for IN (−12.7). The results suggest that when dosed at 5 g/d, IN had a rather negative impact on microbial diversity. In other words, IN specifically increased a limited number of species that outcompeted a larger number of other gut microbes. In contrast, dextran supported the growth of a wide range of gut microbes, as evidenced by the positive value of the combined CMS. 

### 3.6. Dextran Was Selectively Fermented by a Broad Spectrum of Human Gut Microbes Ex Vivo

Dextran and IN affected a broad spectrum of families (Figures S2 and S3). First, both treatments increased *Bifidobacteriaceae* and *Coriobacteriaceae* (<Actinobacteriota), *Bacteroidaceae* (<Bacteroidota), *Acidaminococcaceae*, *Erysipelatoclostridiaceae*, *Erysipelotrichaceae*, *Lachnospiraceae*, *Ruminococcaceae* and *Veillonellaceae* (<Firmicutes), often to various degrees. Dextran increased *Tannerellaceae* (<Bacteroidota), *Methanobacteriaceae* (<Euryarchaeota), *Enterobacteriaceae* and *Sutterellaceae* (<Proteobacteria), while IN decreased their abundance. 

**Figure 5:**
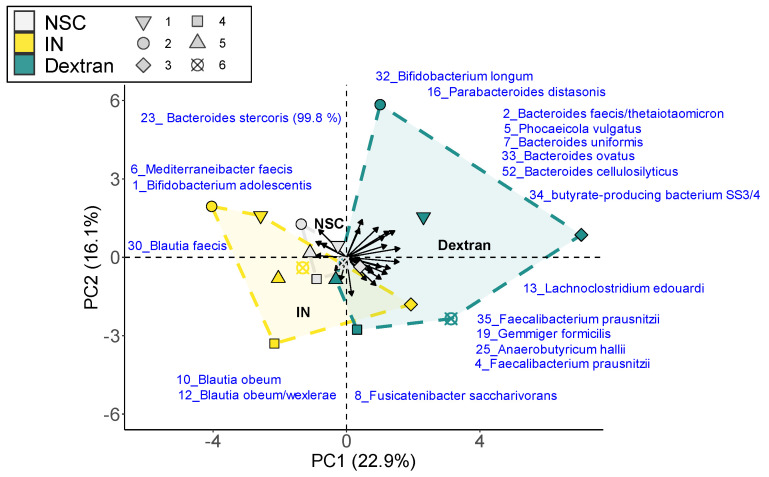
**Dextran and IN stimulated specific human adult gut microbes ex vivo.** The principal component analysis (PCA) summarizes the impact on the gut microbiota. The PCA was based on the standardized abundances of significantly (FDR = 0.2) or consistently affected OTUs by any of the treatments as quantified via 16S rRNA gene sequencing combined with flow cytometry (cells/mL). The different OTUs that underlie this clustering are shown by the arrows and blue text. A detailed representation of the OTUs that significantly increased or decreased upon treatment with the test products is shown in Figure S4.

**Figure 6:**
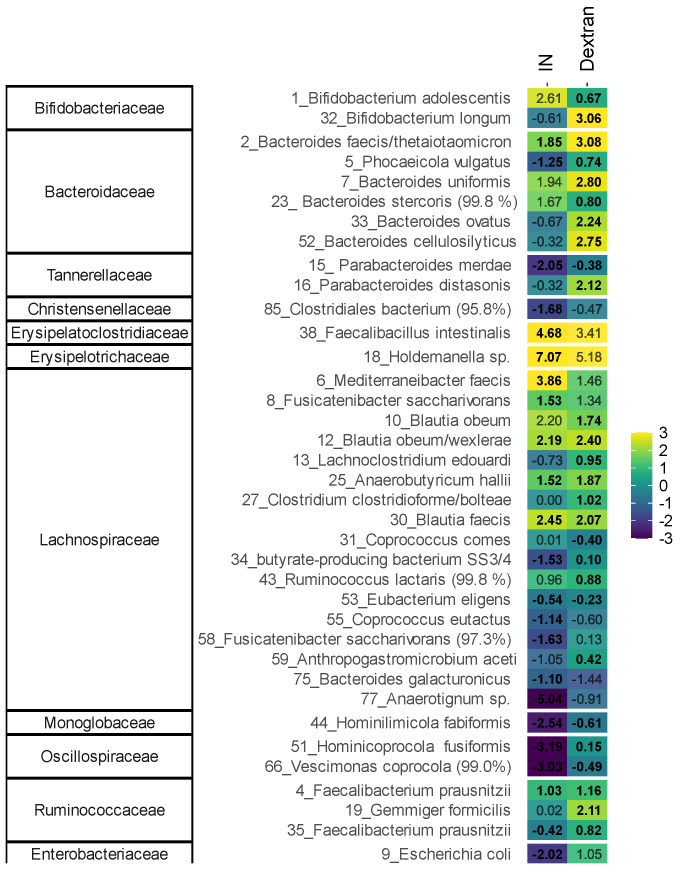
**Dextran and IN affected different OTUs across different OTUs.** Heatmap was generated based on OTUs that were significantly (FDR = 0.20) and non-significantly but consistently affected by dextran and/or IN, expressed as log_2_ (treatment/NSC), averaged over six human adults. Asterisks indicate OTUs that exhibited significant changes upon IN/dextran treatment. Numbers in bold indicate the treatments where significant or consistent changes compared to the NSC occurred. The corresponding families are indicated on the left. A detailed representation of the OTUs that significantly increased or decreased upon treatment with the test products is shown in Figure S4.

To evaluate changes at a higher taxonomic resolution, both exploratory (Figure 5) and in-depth statistical analysis (Figure 6) were performed at the OTU level; 37 OTUs were significantly (FDR = 0.2) or non-significantly but consistently affected by the treatments. The exploratory analysis based on these OTUs indicated that IN and dextran exerted product-specific effects that were consistent across six human adults. IN resulted in a shift to the left along PC1, suggesting treatment effects on OTUs related to *Bacteroides stercoris* (OTU23), *Mediterraneibacter faecis* (OTU6), *Bifidobacterium adolescentis* (OTU1) and *Blautia* spp. (OTU10/12/30). In contrast, dextran resulted in a shift to the right related to the butyrate-producing species SS3/4 (OTU34), *Anaerobutyricum hallii* (OTU25), *Faecalibacterium prausnitzii* OTU35), *Gemmiger formicilis* (OTU19), along with *Bacteroides* spp. (OTU2/7/33/52), *Phocaeicola vulgatus* (OTU5), *Bifidobacterium longum* (OTU32), *Lachnoclostridium edouardi* (OTU13) and *Parabacteroides distasonis* (OT16). 

In-depth statistical analysis demonstrated that dextran significantly or consistently increased a wide range of OTUs (*n* = 22) while lowering levels of a smaller number of OTUs (*n* = 5). In contrast, IN significantly or consistently increased a narrower range of OTUs (*n* = 9) while lowering levels of a larger number of OTUs (*n* = 14). Several of the OTUs negatively affected by IN were promoted by dextran, most notably OTUs related to *Bifidobacterium longum* (OTU32), *Phocaeicola vulgatus* (OTU5), *Parabacteroides distasonis* (OTU16), *Bacteroides ovatus* (OTU33), *Bacteroides cellulosilyticus* (OTU52), *Anthropogastromicrobium aceti* (OTU59) and *Faecalibacterium prausnitzii* (OTU35). This further suggests that dextran supports the growth of a broad spectrum of gut microbes. 

### 3.7. Dextran Similarly Boosted Production of Health-Related SCFA While Inducing Less Gas Production Than IN

**Figure 7:**
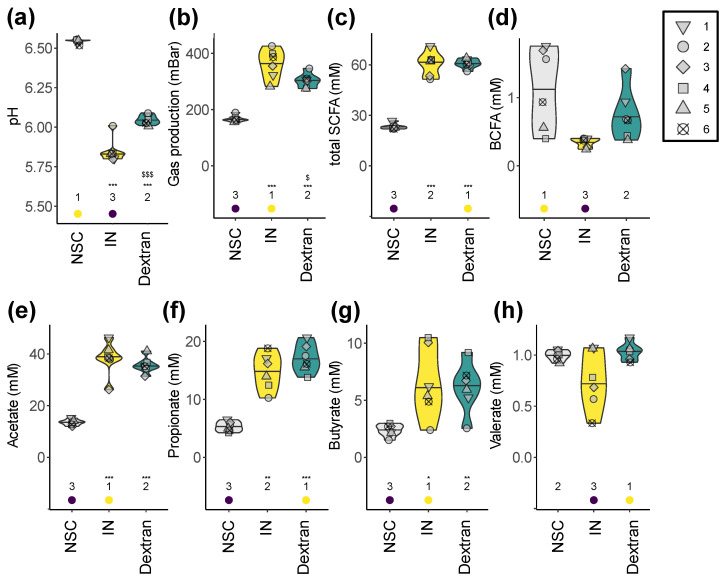
**Dextran similarly boosted the production of health-related SCFA while inducing less gas production than IN.** The impact on (**a**) pH, (**b**) gas production, (**c**) total SCFA, (**d**) bCFA (**e**) acetate, (**f**) propionate, (**g**) butyrate and (**h**) valerate. Statistical differences between treatments and the NSC are indicated with * (0.01 < p_adjusted_ < 0.05), ** (0.001 < p_adjusted_ < 0.01) or *** (p_adjusted_ < 0.001), while differences between dextran and IN are indicated with $/$$$ (0.01–0.05/<0.001). The ranks of the average values per treatment are indicated at the bottom of the figure, with the lowest average being indicated in purple, and the highest value in yellow.

To investigate product-specific effects on metabolite production, key fermentation parameters were recorded (Figure 7). Both IN and dextran increased the production of gases, acetate, propionate, butyrate (and thus, total SCFA), decreased pH and bCFA levels. Importantly, marked differences between IN and dextran were observed. First, while IN tended to most strongly enhance acetate production, dextran more specificity increased propionate (~25% more propionate compared to IN). Additionally, valerate production was markedly reduced upon IN treatment (for four out of six test subjects) compared to dextran. Lastly, gas production was remarkably lower for dextran compared to IN (−31%).

Finally, SCFA production correlated with the presence of specific OTUs (Figure S5), suggesting the involvement of the related species in production of these SCFA upon treatment with IN and/or dextran. First, acetate and propionate correlated with OTUs related to acetate/propionate-producing *Bacteroides* species [9], *Bacteroides uniformis* (OTU7) and especially *Bacteroides faecis/thetaiotaomicron* (OTU2). Acetate production was likely further enhanced by *Bifidobacterium* species [42,43]: *Bifidobacterium adolescentis* (OTU1) for IN and *Bifidobacterium longum* (OTU32) for dextran. Further, butyrate correlated with OTUs related to butyrate-producing species *Anaerobutyricum hallii* (OTU25) [44] and *Faecalibacterium prausnitzii* (OTU4) [45] for both treatments. A notable correlation for particularly dextran was the one between butyrate and *Blautia obeum* (OTU10)/*Gemmiger formicilis* (OTU19). A final remarkable correlation (specific for IN) was the one between acetate/propionate with *Mediterraneibacter faecis* (OTU6).

## 4. Discussion

This study assessed the potential prebiotic effects on gut microbial composition and metabolite production by the high DP dextran NextDext^TM^ compared with the reference prebiotic IN. The ex vivo SIFR^®^ technology was used as this technology has recently been shown to generate insights that are predictive for clinical findings [34]. Treatment effects were assessed for six healthy human adults that covered clinically relevant interpersonal differences, driven by differential levels of *Bacteroidaceae*, *Prevotellaceae* and/or *Ruminococcaceae*, in line with the concept of enterotypes [35,41,46]. Overall, dextran promoted the growth of a broad range of health-related gut microbes, many of which did not increase upon IN supplementation. The effects of dextran were consistent across the six test subjects, stressing that dextran could have predictable effects across different individuals within the population, independently from the initial microbiota composition (or enterotype [41]) of the test subject. Moreover, given the link between enterotypes and transit time, with longer transit times being associated with the *Ruminococcaceae* enterotype [27], dextran might exert effects on microbiota along the entire colon. Altogether, dextran exhibits traits of high-specificity fiber and may beneficially impact gastrointestinal health and beyond. 

Both IN and, to a lesser extent, dextran lowered values of traditional α-diversity indices compared to the NSC, which could be due to two reasons. First, IN and dextran could simply adhere to the prebiotic definition, i.e., upon selective utilization by specific micro-organisms, prebiotics can reduce diversity in favour of this selected number of (beneficial) bacteria [47]. However, a second important aspect, as pointed out by this study, is that the calculation of traditional α-diversity indices ignores differences in cell density and overestimates diversity in low biomass samples (e.g., NSC) compared to high biomass samples (e.g., IN and dextran). To better assess the actual impact of prebiotics (that increase bacterial density) on microbial diversity, the novel CMS was introduced. This CMS is based on quantitative sequencing, and thus, unlike traditional indices that are based on proportional insights, allows us to calculate the number of species that increasingly grew in the presence of a substrate. The CMS is a useful tool for assessing the impact on the microbial diversity by a dietary supplement when evaluated with controlled test models such as the SIFR^®^ technology. Indeed, while dextran already resulted in higher values of traditional α-diversity indices compared to IN, the combined CMS demonstrated that dextran had a positive effect on microbial diversity. In contrast to IN, the number of OTUs supported by dextran largely exceeded the number of OTUs that decreased upon dextran treatment. The stimulation of this broad range of taxa by dextran could originate from its structural properties. While carbohydrates with lower molecular weight and more branches have more non-reducing ends per unit mass and can be more rapidly degraded by exo-acting enzymes produced by selective species such as *Bifidobacterium* spp. [48], high-molecular-weight carbohydrates with fewer branches like dextran are fermented at a slower rate [20,22], and are thus potentially accessible to a wider range of bacteria. Altogether, dextran is a potential type of next-generation dietary fiber that distinguishes itself from established prebiotics by acting as a diversity booster. Overall, the novelty and unicity of this diversity-boosting potential of dextran was thus highlighted by comparing dextran with the reference prebiotic IN that, in contrast, lowered microbial diversity by stimulating specific species while inhibiting many others.

Before linking an increased microbial diversity to potential health benefits, it was of importance to understand the taxa that were responsible for the increased diversity as increased diversity is not necessarily beneficial. For example, during a recent in vitro study with a legacy chemostat gut model, it was stated that encouraging beneficial effects were observed given that the values of a diversity index had increased, even if the underlying data pointed out that the intervention had strongly decreased health-related *Bifidobacterium* spp. at the expense of potentially pathogenic *Enterobacteriaceae* [49]. Such interpretations of diversity indices are problematic; a low diversity of beneficial bacteria is preferred over a high diversity of potentially pathogenic bacteria [50]. As a result, it was of importance to analyse the species that contributed to the diversity boosting effect of dextran.

In agreement with the previous in vitro work [20], dextran strongly increased OTUs related to acetate producing acetate/propionate-producing *Bacteroidaceae* (*B. faecis/thetaiotaomicron*, *B. uniformis*, *B. stercoris*, *B. ovatus*, *B. cellulosilyticus*) [9]. This notable increase likely contributed to the enhanced propionate production in response to dextran, as evidenced by the correlation between propionate levels and propiogenic *B. faecis/thetaiotaomicron* and *B. uniformis* (Figure S5). Further, this effect is specific for dextran, since propionate production was induced 25% more by dextran compared to IN, in line with the significantly greater increase in *Bacteroidaceae*. Intestinal *Bacteroides* has been linked to metabolic health and shown to improve glucose homeostasis, lipid metabolism and promote the production of amino acids associated with vascular health [51,52,53,54,55]. Similarly, propionate was also found to lower serum glucose and decrease cholesterol levels and lipogenesis promoted by acetate [56,57,58]. Interestingly, the acetate level is slightly lower for dextran, compared to IN. Thus, a higher propionate:acetate ratio may indicate more positive effects on lipid storage for dextran [10]. In addition, *Parabacteroides distasonis,* which was supported by dextran, could also alleviate metabolic disorders and obesity in mice by enhancing the production secondary bile acids and succinate, a precursor of propionate [59]. This suggests the potential of dextran in promoting metabolic health and thus its use for weight management.

Interestingly, in contrast to the findings from in vitro batch fermentation [20], ex vivo fermentation of dextran using SIFR^®^ technology also increased the abundance of OTUs related to acetate-producing *Bifidobacteriaceae* (*B. adolescentis*, *B. longum*) and butyrate-producing *Faecalibacterium prausnitzii*. *B. longum* and *B. adolescentis* strains have been developed as probiotics [60] and provide numerous health benefits such as antipathogenic effects, immune modulation, the prevention of gut disorders, and the production of beneficial metabolites and vitamins [61,62,63]. The increase in *B. adolescentis* and *B. longum* highly correlated with increased acetate levels for IN and dextran, respectively (Figure S5). This enhanced acetate production boosted the production of butyrate by *F. prausnitzii* and *A. hallii* via cross-feeding [11,12,13,64]. This is further confirmed by the positive correlation between OTUs related to *F. prausnitzii* and *A. hallii* with the high butyrate levels. Other notable taxa that potentially contributed to high butyrate levels for dextran were *Fusicatenibacter saccharivorans*, *Clostridium clostridioforme/bolteae* and known butyrate producers *Blautia obeum/wexlerae* [65] and *Gemmiger formicilis* [66]. Butyrate has strong anti-inflammatory effects and plays a vital role in promoting gastrointestinal health. It is a key energy source of colonic epithelium, regulates the expression and assembly of tight junction proteins and thus improves gut barrier integrity [67,68,69]. Further, butyrate increases energy expenditure and insulin sensitivity and thus has therapeutic potential in weight control and treatment of type-2 diabetes [70,71,72]. Thus, strains of potent butyrate producers *Faecalibacterium prausnitzii* and *Anaerobutyricum hallii* have been studied for their probiotic effects [71,73,74]. In addition, anti-microbial and anti-inflammatory effects, also beyond the gut, were previously found for the dextran-induced *Parabacteroides distasonis,* which could alleviate colitis and rheumatoid arthritis in mice [75,76,77]. Overall, the diverse profile of health-related gut bacteria supported by dextran, and related metabolite production demonstrate great potential in improving gastrointestinal health and beyond.

Importantly, while total health-related SCFA production was similar for dextran and IN, gas production was significantly lower for dextran (−31%) compared to IN, consistent with previous in vitro work [20]. The chemistry of the prebiotic and the composition of the microbiota were found to be relevant for microbial gas production [78]. A strong gas production upon intake of IN (or other fructans) due to rapid colonic fermentation could result in limited tolerance at high doses [79,80]. In contrast, fermentation of glucooligosaccharides was found to generate markedly less gas than fructo- and galactooligosaccharides [81]. Excessive gas production caused by consumption of non-digestible fibers is associated with abdominal symptoms such as bloating, constipation, belching and abdominal pain [82]. The lower gas production suggests that dextran may be better tolerated than IN, while still achieving comparable beneficial metabolite production. 

Finally, while the extent of the increase was minor, valerate levels tended to be higher for dextran compared to the NSC and particularly IN. While valerate is much less studied than the other SCFA, it has also been demonstrated to decrease the growth of cancer cells [83] or to exert antipathogenic effects against *C. difficile* [84].

In conclusion, besides its other industrial applications, the high DP dextran NextDext^TM^ exhibits traits of a high-specificity prebiotic, consistently supporting a broad spectrum of health-related gut bacteria across different donors with different enterotypes. Dextran did not only show strong bifidogenic effects but also increased other health-related species belonging to various taxa and thus may have a positive impact on gastrointestinal health and beyond. While the potential benefits of dextran make it a highly promising substrate, future clinical studies will further help to elucidate whether its application should be preventative or curative. Based on the findings of the current study, these clinical studies could target patients suffering from, amongst others, metabolic disease (overweight, obesity), constipation, and even cognitive and neurodegenerative diseases.

## Data Availability

The datasets generated during and/or analysed during the current study are available from the corresponding author upon reasonable request.

## Supplementary Materials

The following supporting information can be downloaded at: https://www.mdpi.com/article/10.3390/biology13010051/s1, Figure S1: Increased bacterial cell densities increase the limit of detection (LOD) of quantitative 16S rRNA gene profiling. Figure S2: Dextran and IN affected different bacterial families across different phyla. Figure S3: Dextran and IN affected different bacterial families. Figure S4: Dextran and IN affected different OTUs. Figure S5: Dextran and IN exerted stimulatory effects on a range of OTUs that correlated with the production of specific SCFAs.
