# Supplementary material for: A Long-Chain Dextran Produced by Weissella cibaria Boosts the Diversity of Health-Related Gut Microbes Ex Vivo
Source: Biology (Basel). 2024 Jan 18;13(1):51. doi: 10.3390/biology13010051 (PMC10813514; doi:10.3390/biology13010051)
Supplement: Supplementary file 1 [file biology-13-00051-s001.zip › biology-2724018-supplementary.pdf]

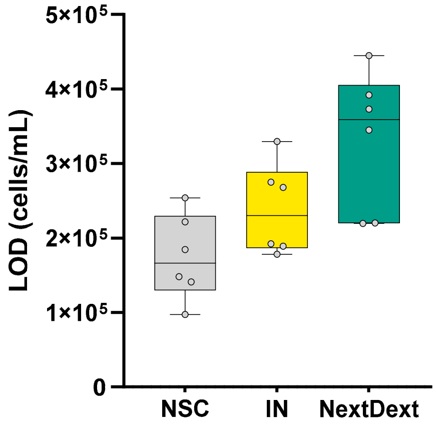

Figure S1. Increased bacterial cell densities increase the limit of detection (LOD) of quantitative 16S rRNA gene profiling. LOD for NSC, IN and dextran (NextDext™) samples was calculated using the formula $LOD = \frac{1}{total\ reads} \times Bacterial\ cell\ density$. Bacteria with cell density under the LOD in the respective samples could not be detected by 16S rRNA gene sequencing. Samples were collected after 24 h of simulated colonic incubations.

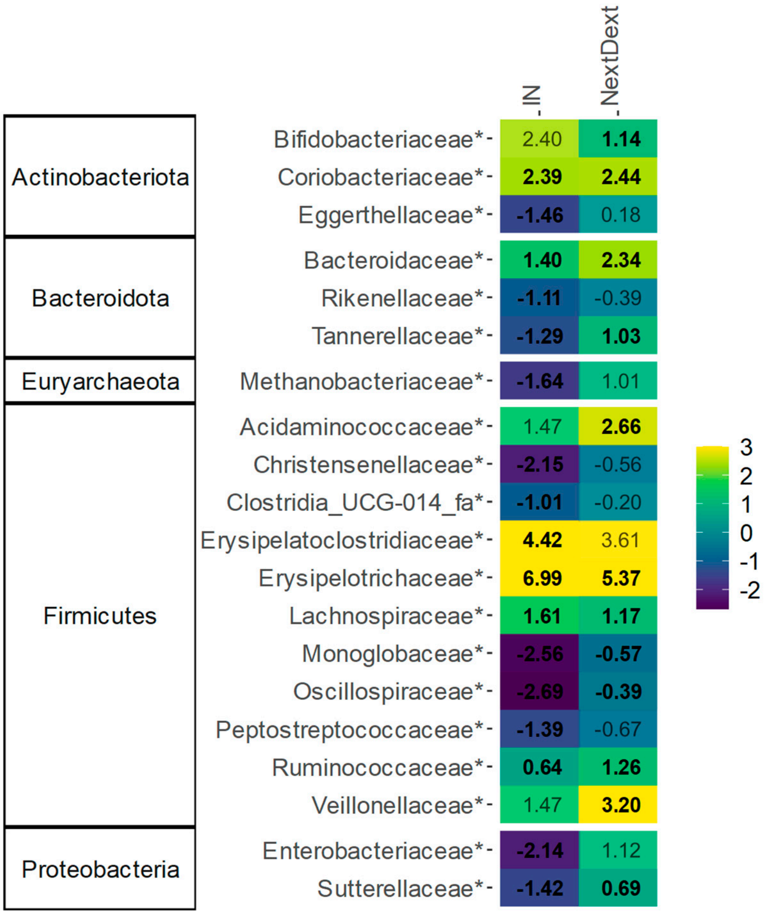

Figure S2. Dextran (NextDext™) and IN affected different bacterial families across different phyla. Heatmap was generated based on families that were significantly (FDR = 0.20) and non-significantly but consistently affected by any of the treatments, expressed as log_2 (treatment/NSC), averaged over six human adults at 24 h, as tested with the *ex vivo* SIFR® technology. Asterisks indicate families that exhibited significant changes upon IN/dextran treatment. Numbers in bold indicate the treatments where significant or consistent changes compared to the NSC occurred. The corresponding phyla are indicated on the left.

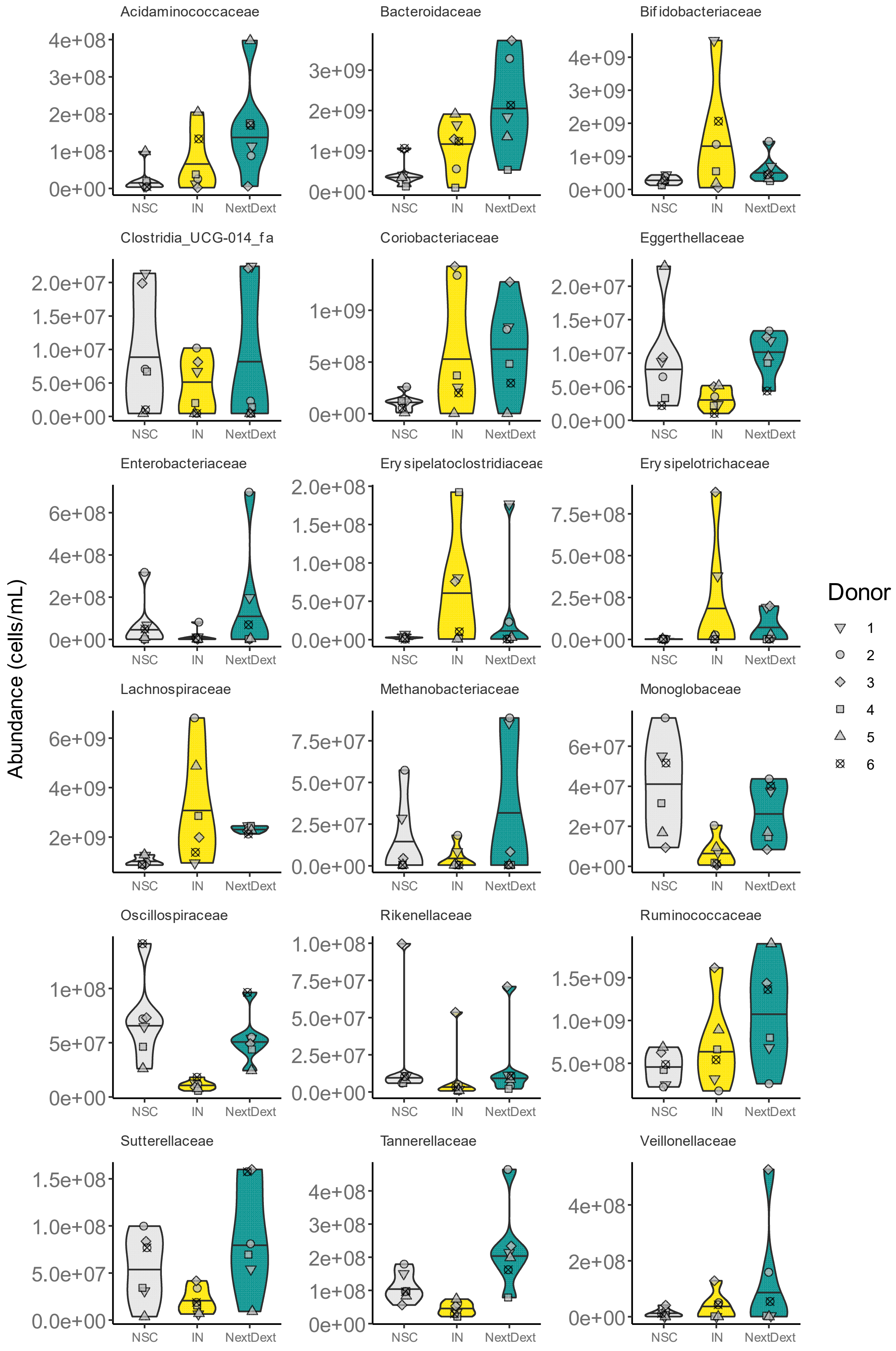

Figure S3. Dextran (NextDext™) and IN affected different bacterial families. The impact on the significantly (FDR = 0.2) and non-significantly but consistently affected families, presented in Figure S3.

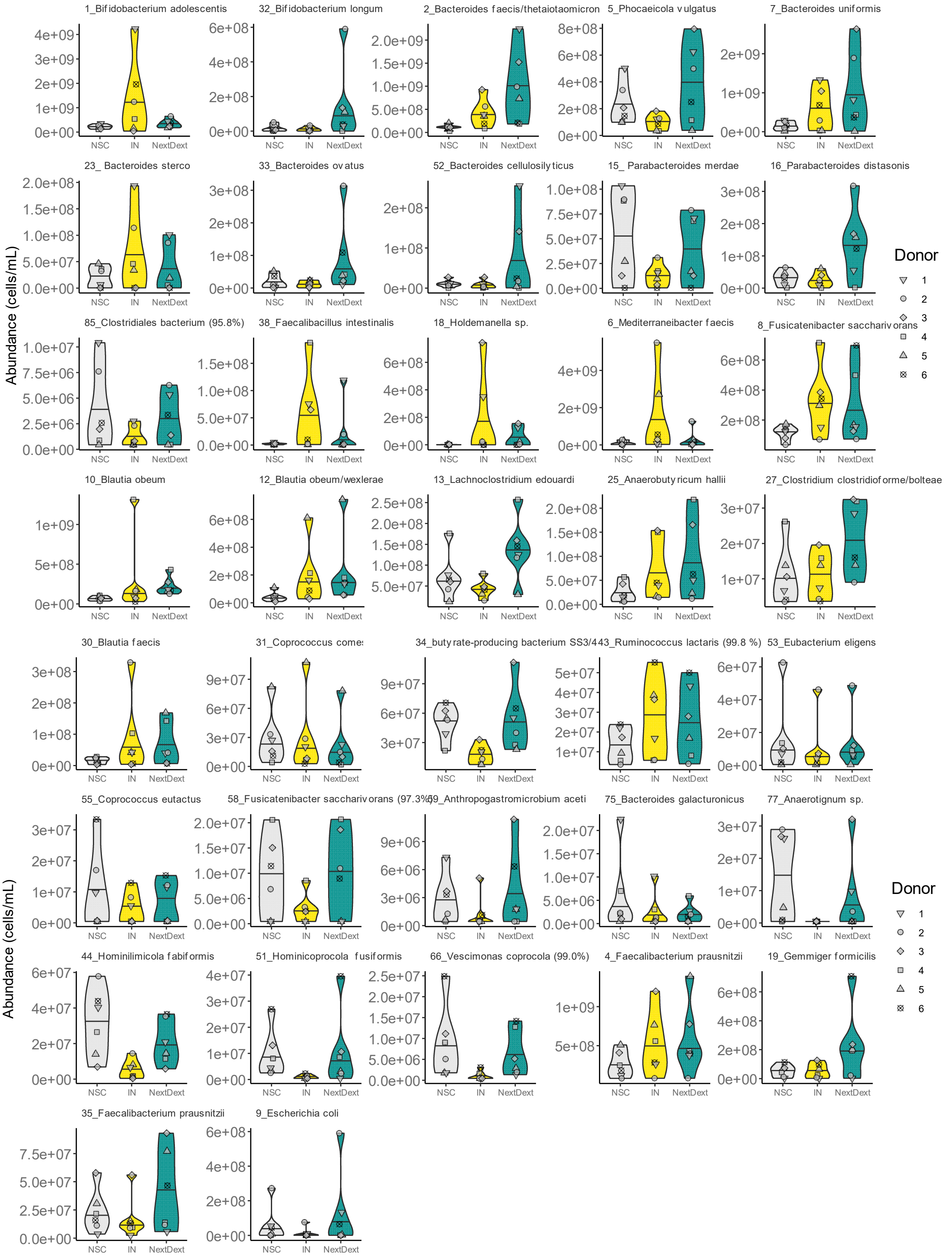

Figure S4. Dextran (NextDext™) and IN affected different OTUs. The impact on the significantly (FDR < 0.2) and non-significantly but consistently affected OTUs, presented in Figure 5.

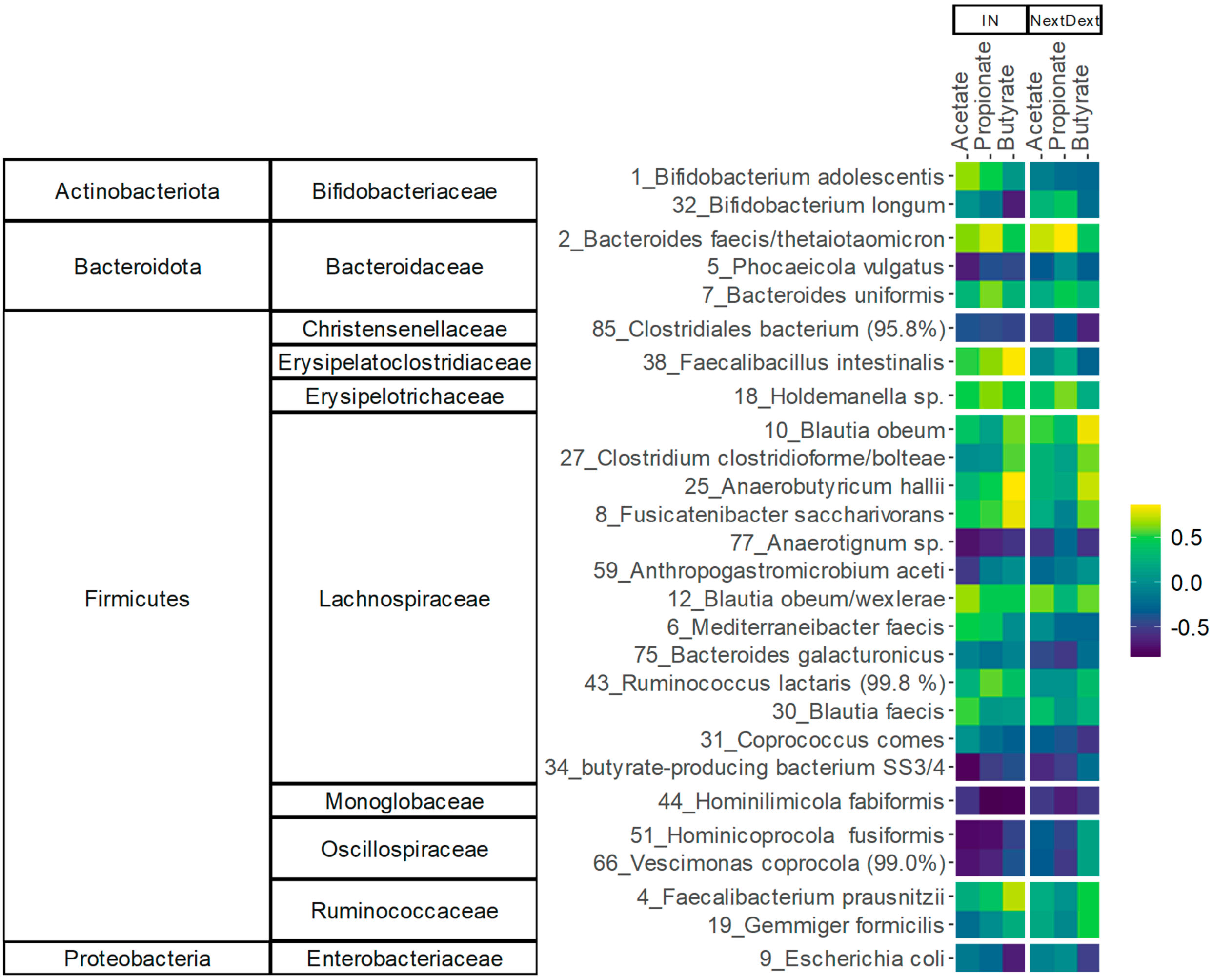

Figure S5. Dextran (NextDext™) and IN Exerted Stimulatory Effects on a range of OTUs that correlated with production of specific SCFAs. Heatmap showing correlations based on a regularized canonical correlation analysis (rCCA) between acetate, propionate, butyrate and OTUs that were significantly or consistently affected by dextran and IN (presented in Figure 5), as tested with the *ex vivo* SIFR® technology.
